# Hemoglobinopathy diagnosis in LMICs: from hematological screening to molecular confirmation

**DOI:** 10.3389/fgene.2026.1969093

**Published:** 2026-11-02

**Authors:** Savitri M. Nerune, Sayandeep K. Das

**Affiliations:** Department of Pathology, Shri B. M. Patil Medical College Hospital and Research Centre, BLDE (Deemed to be University), Vijayapura, Karnataka, India

**Keywords:** cascade screening, genetic counseling, hemoglobinopathies, high-performance liquid chromatography, low- and middle-income countries, molecular diagnosis, sickle cell disease, thalassemia

## Abstract

Hemoglobinopathy diagnosis in low- and middle-income countries (LMICs) is limited not only by access to technology but also by attrition between screening, phenotypic resolution, molecular confirmation, counseling, and linkage to care. India illustrates this problem: national guidance distributes screening, high-performance liquid chromatography (HPLC), and molecular services across district, regional, and tertiary levels, making inter-facility referral intrinsic when advanced testing is unavailable locally. Families may consequently face test charges, travel, wage loss, repeat sampling, delayed results, and incomplete partner or family testing. This Mini Review integrates evidence from India with diagnostic models from Bangladesh, Sri Lanka, Thailand, Indonesia, and Ghana. We examine hematological triage, the limitations of HPLC and electrophoretic methods, indications for selective molecular testing, and the structural, financial, and sociocultural causes of late or incomplete diagnosis. We propose a three-level diagnostic model supported by standardized interpretive reporting, specimen networks, active recall, local-language counseling, and measurable completion indicators. The objective is not universal sequencing, but reliable conversion of every abnormal screen into an actionable individual and familial diagnosis, supported by cascade screening and selective molecular confirmation of rare or complex genotypes that cannot be resolved phenotypically cannot be resolved phenotypically.

## 1 Introduction

Hemoglobinopathies comprise qualitative and quantitative defects of globin-chain synthesis and structural hemoglobin variants. Thalassemias and sickle cell disorders collectively impose a major burden across South and Southeast Asia and sub-Saharan Africa, where endogamy, consanguinity, migration, malaria-related selection, and population-specific founder variants produce marked genetic heterogeneity (Harteveld et al., 2022; Goh et al., 2020; Twum et al., 2023). India contains overlapping geographic signatures: HbE is concentrated in the northeast and eastern regions, HbS extends across central, western, eastern, and parts of southern tribal belts, HbD-Punjab is enriched in the northwest, and beta-thalassemia occurs across multiple communities (Sinha et al., 2009; Colah et al., 2015; Thaker et al., 2024). Consequently, similar hematological phenotypes may represent carrier states, clinically significant disease, compound heterozygosity, or co-inherited modifiers with different clinical and reproductive implications.

Diagnosis usually begins with clinical evaluation, complete blood count (CBC), red-cell indices, peripheral smear, and high-performance liquid chromatography (HPLC). However, borderline HbA2, alpha-thalassemia, rare or co-eluting variants, history of transfusion, infancy, iron deficiency and treatment effects may prevent definitive assignment (Colaco and Nadkarni, 2021; Sani et al., 2024). Family cascade screening and molecular testing can resolve such uncertainty, but in LMICs their value is constrained by centralized services, cost, transport, delayed turnaround, and failure to test partners or relatives.

We define the diagnostic completion gap as failure of a screen-positive individual to reach an actionable interpretation, documented communication and counseling, and the next clinically or reproductively indicated step. The review used PubMed/MEDLINE, official LMIC program documents, and reference-list screening. The core search ended on 3 July 2026. Methods are provided in Supplementary Material 1. This is a focused narrative evidence synthesis rather than a systematic review or meta-analysis, and the included examples are illustrative rather than exhaustive. No unpublished institutional data were included, and extrapolations are identified as author inferences or proposals.

## 2 Regional heterogeneity and diagnostic architecture

Diagnostic pathways must reflect regional variant spectra and health-system architecture. Indian guidance assigns carrier confirmation to district laboratories and DNA analysis or prenatal diagnosis to regional and tertiary centers, making referral intrinsic when advanced testing is unavailable locally (Ministry of Health and Family Welfare, 2016). Remote-area Indian work documented transport of specimens to laboratories in larger cities, while newer evidence confirms that whole-blood collection, storage, and temperature control constrain population-scale HPLC (Colah et al., 2010; Jena et al., 2025). Taken together, these sources suggest that some tier 2 and tier 3 services depend on courier referral or scheduled batches of individually identified samples. Although batching may improve analyzer utilization, it can prolong turnaround time. Diagnostic capacity therefore depends on reagents, maintenance, quality assurance, interpretive expertise, specimen logistics, and reliable result return, not instrument installation alone.

Table 1 synthesizes geographic variation, diagnostic processes, numerical implementation signals, and completion bottlenecks across selected LMIC settings. Ghana demonstrates both reach and fragility: its reference laboratory reported 528,735 newborns screened during 1995–2020, with 9,068 sickle cell disease diagnoses (1.71%), but funding constrained geographic coverage (Noguchi Memorial Institute for Medical Research, n.d.). At Korle Bu, initial screening reached 4,427 of 9,990 eligible newborns (44.3%); workflow redesign increased coverage to 83.7%, although staffing, supply, and result delays persisted (Segbefia et al., 2021). Other models expose different handoffs: Bangladeshi carrier frequency varied from 4.2% to 27.1% by division; Sri Lanka continued to record 45–55 new beta-thalassemia births annually; 46.4% of eligible Thai male partners were not screened; and West Javan cascade screening identified carriers in 42.7% of index-family relatives versus 10.6% of controls (Noor et al., 2020; Premawardhana et al., 2019; Malasai et al., 2026; Susanah et al., 2022).

**TABLE 1:** Regional hemoglobinopathy signatures, diagnostic processes, and completion bottlenecks in selected LMIC settings.

Setting and representative signal	Geographic/variant pattern	Diagnostic process illustrated	Completion bottleneck and interpretation
India 33,270 women screened across 36 districts in five states; 2,268 carriers (6.8%). Partner testing: 1,959/2,268 (86.4%). Fetal testing: 246/339 at-risk couples (72.6%). Approximate program cost: INR 550 per enrolled family	HbE is concentrated in the northeast and east; HbS across central, western, eastern, and parts of southern tribal belts; HbD-Punjab in the northwest; beta-thalassemia occurs nationwide. State-level carrier rates in the implementation study ranged from 2.9% to 11.0%	Maternal HPLC (→) partner HPLC (→) stepwise qPCR/Sanger/gap-PCR/MLPA/NGS (→) fetal testing. Eighty-seven clinics used barcoding, cold-chain courier, hub-and-spoke laboratories, coordinators, and a digital dashboard	Centralization can deliver scale only when logistics are treated as part of diagnosis. Four percent of enrolled women did not complete testing; 1,182 samples were rejected because of deterioration or mislabeling and 195 were lost during collection/transport. Twenty fathers worked in other cities. Tier-2/3 referral pathways therefore require tracked transport, active recall, and funded counseling rather than passive advice (Agarwal et al., 2025; Ministry of Health and Family Welfare, 2016)
Ghana National reference-laboratory data: 528,735 newborns screened during 1995–2020; 9,068 SCD diagnoses (1.71%). Korle Bu implementation: 4,427/9,990 eligible newborns screened (44.3%), 79 presumptive SCD (1.8%), with workflow redesign increasing coverage to 83.7%	High HbS burden with marked service-level variation. National screening used DBS and IEF, but limited funding restricted geographic coverage largely to Ashanti until 2020, when 14 sites were added to the original 10	DBS collection (→) centralized IEF at the national laboratory (→) confirmatory classification (→) parental education and linkage to care. Site-level workflow redesign addressed recruitment and collection failures	Coverage, confirmation, and linkage must be audited separately. Inadequate staffing, supply shortages, delayed results, funding limitations, weak records, migration, and stigma can interrupt the pathway; 66% of a separate SCD cohort were diagnosed during a pain crisis (Noguchi Memorial Institute for Medical Research, n.d.; Segbefia et al., 2021; Sims et al., 2021; Twum et al., 2023)
Bangladesh Nationwide survey, n = 1,877. Carriers of abnormal beta-globin genes: 11.89%; HbE trait: 8.68%; beta-thalassemia trait: 2.24%. Carrier frequency ranged from 4.2% in Khulna to 27.1% in Rangpur. 62.3% lacked prior etiologic or severity knowledge	HbE predominates, with strong divisional heterogeneity. Carrier frequency was 23.5% among participants with consanguineous parents versus 11.4% among those without	CBC and capillary electrophoresis with ferritin assessment; high-resolution melting for common variants and Sanger sequencing for unresolved samples	Uniform national screening thresholds can conceal high-risk divisions and ethnic groups. Geographic targeting must be combined with public education and molecular resolution of equivocal phenotypes; consanguinity modifies risk but does not identify all carriers (Noor et al., 2020)
Sri Lanka Nationwide hospital survey, n = 1,774 across 23 centers: homozygous beta-thalassemia 68.7%; HbE/beta-thalassemia 20.3%. Estimated 45–55 new beta-thalassemia births annually, without a clear 19-year decline. One-stop screening cohort, n = 1,324	Disease burden is island-wide but center volume and expertise vary. Beta-thalassemia and HbE/beta-thalassemia dominate the treated population	Low red-cell indices or OF/DCIP triage followed by capillary electrophoresis. In the one-stop model, low indices had 100% sensitivity and 68.4% specificity; OF plus DCIP had 100% sensitivity and 77.8% specificity against capillary electrophoresis	A national network does not guarantee prevention when counseling, partner testing, and prenatal action are inconsistent. Expert hubs should support peripheral screening, standardized interpretation, and outcome tracking rather than simply receive samples (Allen et al., 2019; Premawardhana et al., 2019)
Thailand 18,976 pregnancies screened; 12,499 maternal screens were positive. Complete partner data were available for 6,698 couples; 1,226 (18.3%) were high risk. Severe disease was diagnosed in 236 fetuses (1.24% of all screened pregnancies)	Alpha-thalassemia and HbE/beta-thalassemia are major reproductive risks; severe fetal outcomes include Hb Bart hydrops fetalis, homozygous beta-thalassemia, and beta-thalassemia/HbE	Maternal MCV/MCH and hemoglobin typing → partner testing → molecular characterization → prenatal diagnosis for high-risk couples	The major failure point was partner completion: 46.4% of eligible male partners were not screened. Sequential maternal-first designs should be replaced, where feasible, by simultaneous couple sampling, active tracking, or a same-visit partner plan (Malasai et al., 2026)
Indonesia (West Java) Extended-family screening: 150 relatives of index families versus 151 control-family relatives. Carriers were detected in 42.7% versus 10.6%, respectively (p < 0.001)	Detected states included beta-thalassemia trait, beta-thalassemia/HbE, suspected alpha-thalassemia, and beta-globin variants	Hb, MCV, MCH, and peripheral smear triage (→) hemoglobin analysis in screen-positive relatives (→) counseling	When population screening is sporadic, an index case can become a high-yield entry point. Cascade screening should be operationalized through pedigrees and scheduled family testing, not delegated entirely to verbal disclosure by the index family (Susanah et al., 2022)
Southeast Asia overview Meta-analysis of 29 studies involving 83,674 participants: pooled alpha-thalassemia prevalence 22.6%. Country estimates included Vietnam 51.5%, Cambodia 39.5%, Laos 26.8%, Thailand 20.1%, and Malaysia 17.3%; heterogeneity exceeded 99.5%	Marked ethnic and malaria-belt heterogeneity; cis alpha-zero deletions are clinically important because at-risk couples can conceive fetuses with Hb Bart hydrops fetalis	Protein-based methods and adult fractions may be non-diagnostic for alpha-thalassemia; regionally appropriate deletion testing and selective sequencing are required when suspicion persists	Pooled national averages should not be converted into a single screening rule. Mutation panels, cut-offs, and counseling priorities must be validated against local subpopulations and referral catchments (Goh et al., 2020)

Values arise from different populations, years, denominators, and study designs and should not be interpreted as directly comparable national prevalence estimates. Carrier frequency, screening coverage, and completion of indicated testing represent distinct outcomes and are not pooled. Percentages were recalculated only from published numerators and denominators. CBC, complete blood count; CE, capillary electrophoresis; DBS, dried blood spot; HPLC, high-performance liquid chromatography; IEF, isoelectric focusing; SCD, sickle cell disease; OF, osmotic fragility; DCIP, dichlorophenolindophenol; qPCR, quantitative polymerase chain reaction; MLPA, multiplex ligation-dependent probe amplification; NGS, next-generation sequencing; Hb, hemoglobin; MCV, mean corpuscular volume; MCH, mean corpuscular hemoglobin; PCR, polymerase chain reaction; INR, Indian rupees; LMICs, low- and middle-income countries.

## 3 Hematological screening: a high-value entry point

Hematological screening is indispensable because it is comparatively affordable and widely deployable. Suspicion should be raised by unexplained microcytosis, anemia that persists despite adequate iron therapy, hemolysis, jaundice, splenomegaly, recurrent pain, an affected relative, or an adverse reproductive history. The minimum first-line package is a CBC with mean corpuscular volume (MCV), mean corpuscular hemoglobin (MCH), red-cell count, and red-cell distribution width (RDW), peripheral smear, reticulocyte count where available, and assessment of iron status when indicated (Sani et al., 2024).

Low MCV and MCH with a relatively preserved or increased red-cell count suggest thalassemia trait, but overlap with iron deficiency is substantial, particularly in pregnancy and childhood. Discriminant formulae can support triage but are affected by age, inflammation, pregnancy, analyzer platform, iron deficiency, and local mutation spectrum. Regional work from our group showed that combined erythrocyte and reticulocyte parameters improve discrimination between iron deficiency anemia and thalassemia trait, but no index can replace hemoglobin fractionation (Nerune et al., 2024). The naked-eye single-tube red-cell osmotic fragility test (NESTROFT), dichlorophenolindophenol (DCIP) testing, sickling tests, solubility tests, and validated point-of-care tests point-of-care tests can increase reach when selected according to local disease distribution (Gosavi et al., 2021).

The principal danger is using screening rules as exclusion rules. Structural-variant carriers may have normal indices, and silent beta variants or alpha-thalassemia may escape conventional cut-offs. In Bangladesh, apparently normal indices occurred in some HbE and beta-thalassemia carriers (Noor et al., 2020). Indian antenatal evidence likewise shows that restrictive CBC thresholds can omit clinically relevant variants (Singh et al., 2023). Screening should therefore stratify probability, not declare genotype. Persistent clinical suspicion, abnormal indices, a positive variant-directed screen, or discordant findings should trigger definitive phenotyping.

## 4 Phenotypic resolution: strengths, grey zones, and recurrent pitfalls

Cation-exchange HPLC and capillary electrophoresis (CE) can be used as alternative primary hemoglobin-fractionation platforms in many routine settings. These methods, conventional electrophoresis, and isoelectric focusing (IEF) may provide complementary or orthogonal information when an initial pattern is ambiguous or potentially affected by co-elution. HPLC quantifies major fractions; CE separates by electrophoretic mobility and may resolve HPLC co-elution; electrophoresis supplies orthogonal information; and IEF is valuable in newborn screening. An analyzer-defined retention or migration window is an analytical zone, not a molecular identity. Interpretation must integrate peak morphology, fraction size, CBC, age, pregnancy, transfusion or treatment, phenotype, and family data (Sani et al., 2024; Thaker et al., 2024).

Borderline HbA2 is a consequential grey zone. Mild or silent *HBB* variants, co-inherited alpha- or delta-thalassemia, iron deficiency, pregnancy, age, population background, and platform bias may move results across a fixed threshold. Iron deficiency generally lowers HbA2 in non-carriers, but its effect in beta-thalassemia carriers is inconsistent (Colaco and Nadkarni, 2021; Thilakarathne et al., 2024). In an Indian cohort of 205 borderline cases, 168 had an identifiable genetic or acquired explanation; some beta-thalassemia carriers had HbA2 below 3.5% with MCV above 80 fL (Colaco et al., 2022).

HbA2 is a context- and platform-dependent measurement, not a binary genotype marker. Pregnancy-specific intervals may be lower than non-pregnant intervals, whereas megaloblastic anemia, hyperthyroidism, and some antiretroviral regimens may produce borderline or falsely elevated values. Co-eluting variants can create apparent increases without increased delta-chain synthesis. Supplementary Table S1 summarizes key biological and analytical scenarios and confirmatory responses (Kang et al., 2022; Sahoo et al., 2023; Kaur et al., 2023; Thilakarathne et al., 2024).

Other pitfalls include donor-derived mixed fractions after transfusion, physiologically high HbF and low HbA2 in infancy, hydroxyurea-associated changes, apparently normal adult fractions in alpha-thalassemia, delta-chain variants splitting or reducing the HbA2 fraction, and compound heterozygosity. Difficulty distinguishing HbSS from HbS/beta-thalassemia should prompt review of HbA, HbA2, indices, treatment history, and parental samples; a severe phenotype reported as “trait” requires immediate reassessment (Yadav et al., 2026).

We propose the explicit category “indeterminate hemoglobinopathy pattern” rather than forced identification. Reports should state the source of uncertainty, prioritized differentials, the recommended orthogonal or molecular test, whether parental samples could establish segregation or partner testing could clarify reproductive risk, and urgency in infancy or pregnancy. This converts analytical uncertainty into an actionable referral instruction.

## 5 Selective molecular confirmation

Molecular testing should be the capstone of a tiered pathway, not a substitute for careful hematology and protein analysis. Referral is justified when microcytosis persists after iron deficiency has been addressed despite normal or non-diagnostic HbA2; HbA2 is borderline in either member of an at-risk couple; clinical, hematological, HPLC, and CE findings disagree; an unidentified or co-eluting peak is present; HbSS cannot be distinguished from sickle beta-thalassemia; transfusion, infancy, or treatment prevents reliable phenotyping; apparent carrier status conflicts with disease severity; or precise parental variants are required for prenatal, preimplantation, or recurrence-risk assessment (Sabath, 2023; Yadav et al., 2026).

The assay should match the suspected lesion and regional spectrum. Locally validated allele‐specific polymerase chain reaction (PCR), amplification refractory mutation system PCR (ARMS‐PCR), or reverse dot blot can resolve common *HBB* variants; gap-PCR is efficient for prevalent alpha-globin deletions; multiplex ligation-dependent probe amplification (MLPA) detects copy-number changes and larger rearrangements; Sanger sequencing addresses focused single-gene questions; and targeted next-generation sequencing (NGS) is appropriate after common panels are negative or a complex genotype is suspected (Achour et al., 2021). NGS is not intrinsically definitive: *HBA1*/*HBA2* homology, copy-number detection, bioinformatic capacity, variants of uncertain significance, cost, and the need for segregation or orthogonal confirmation remain important limitations.

In resource-constrained settings, the relevant question is not whether every patient can be sequenced, but which validated molecular test is most appropriate and cost-effective for resolving the clinically or reproductively consequential uncertainty, considering expected diagnostic yield and the suspected variant spectrum. Regionally updated mutation panels can often provide greater diagnostic value than indiscriminate broad sequencing.

## 6 Why diagnosis remains late or incomplete

Late diagnosis follows absent or incomplete newborn screening, variable childhood phenotypes, and repeated attribution of microcytosis to nutritional iron deficiency without reassessment (Twum et al., 2023). Episodic care for anemia, jaundice, infection, or pain may proceed without longitudinal records, while limited interpretive expertise, unavailable family data, and transfusion before definitive testing further obscure classification. Ghana shows the distance between program existence and completion: funding constrained expansion of its longstanding DBS-IEF program, and one tertiary-site study initially screened only 44.3% of eligible newborns (Noguchi Memorial Institute for Medical Research, n.d.; Segbefia et al., 2021). In a separate cohort of 354 people with sickle cell disease, only 44% had been diagnosed by 4 years and 66% were diagnosed during a pain crisis (Sims et al., 2021). Adult and pregnancy case reports from India and other LMICs illustrate missed opportunities but cannot estimate their prevalence (Sharma et al., 2020).

In India, instrument availability is necessary but not sufficient. National guidance identifies sustained financing, district confirmation laboratories, regional DNA services, quality systems, and trained personnel as core requirements (Ministry of Health and Family Welfare, 2016). A survey of 38 centers in 20 countries likewise documented wide variation in genomic access and laboratory policies (Stephanou et al., 2026). In a Gujarat study of 1,900 samples, borderline HbA2, unknown peaks, and complex HbA2/HbF patterns required clinical, red-cell, and family correlation rather than automated window assignment (Anandani et al., 2025). The 2025 national sickle-cell application includes a “Confirmatory Test List” for positive solubility tests lacking HPLC or electrophoresis results, operationalizing the screen-to-confirmation gap (National Informatics Centre and National Health Mission, 2025). We infer that low-volume laboratories are particularly vulnerable when maintenance, external quality assessment, specialist interpretation, referral logistics, and result tracking cannot be sustained.

Affordability is therefore a cumulative pathway cost. Beyond CBC, HPLC, or DNA fees, families may pay for intercity travel, sample transport, repeat collection, accommodation, wage loss, partner testing, result retrieval, counseling, and prenatal procedures. In a recent Indian antenatal program, 4% of enrolled women did not complete testing, largely because samples deteriorated, were mislabeled, or were lost during transport; 20 fathers worked in other cities, and only 72.6% of at-risk couples completed fetal testing (Agarwal et al., 2025). Even when the program-level cost was approximately INR 550 per enrolled family, completion depended on courier networks, coordinators, digital dashboards, counseling, and transport support.

From an implementation-science perspective, we propose that diagnostic underinvestment also suppresses regional research and development. The wider LMIC rare-disease literature identifies high genomic costs, underrepresentation of local populations, and limited locally generated variant data as linked barriers to both diagnosis and research (Gonzaga-Jauregui et al., 2025). The Sickle Pan-African Research Consortium similarly treated skills in care, data management, and research as interdependent, training 1,726 participants across Ghana, Nigeria, and Tanzania (Nnodu et al., 2022). Taken together, these findings suggest that laboratories without stable case volumes, quality systems, biobanking, interoperable data, and molecular infrastructure are less able to validate local HbA2 thresholds, refine mutation panels, characterize rare variants, or prospectively measure stage-specific attrition. This is an evidence-informed author inference rather than a measured national effect. Service strengthening and research capacity should therefore be treated as mutually reinforcing rather than competing investments.

## 7 Counseling, partner testing, family screening, and consanguinity

A laboratory result does not complete diagnosis. Carrier status has limited preventive value unless understood, disclosed safely, and connected to partner, family, and reproductive services. Counseling should explain autosomal inheritance, distinguish carrier state from disease, communicate residual uncertainty when molecular testing is incomplete, and address risks to partners, siblings, and extended relatives. Options must remain voluntary, confidential, and non-directive (Ministry of Health and Family Welfare, 2016).

Where genetic counselors are scarce, counseling may collapse into report delivery. Across 36 districts in five Indian states, 33,270 pregnant women were screened and 2,268 carriers identified; 86.4% of partners were tested, but only 246 of 339 at-risk couples completed fetal testing (Agarwal et al., 2025). Refusal, unreachable partners, gestational delay, miscarriage, and partners working elsewhere contributed to attrition. Transport expense, work absence, misconceptions, stigma, delayed index diagnosis, and failure to communicate risk also impede cascade testing (Pandharipande et al., 2025). Structured pre- and post-counseling assessment is preferable to one-time information delivery (Samanta et al., 2024).

Consanguinity and endogamy are related but not synonymous. Consanguinity increases the probability that partners share ancestral alleles; endogamy can maintain high variant frequencies without close biological relatedness. Neither is the sole cause of disease, and non-consanguineous couples remain at risk in high-carrier populations. In Bangladesh, carrier frequency was 23.5% among participants with consanguineous parents and 11.4% among those without, supporting population-appropriate rather than ancestry-only testing (Noor et al., 2020).

We recommend a completion-oriented sequence: explain the result; verify understanding by teach-back; construct a three-generation pedigree; identify eligible partners and first-degree relatives; provide a local-language family communication sheet; arrange testing rather than merely advise it; document acceptance, refusal, or access failure; and repeat counseling at pregnancy planning or early antenatal registration.

Several countries identify carriers earlier. India’s National Sickle Cell Anaemia Elimination Mission combines premarital and preconception screening and genetic counseling with population screening from birth to 18 years (incrementally to 40 years) and school- or college-based early detection in high-prevalence areas (National Health Mission, Government of India, 2026). Malaysia has nationally screened Form 4 students, generally aged 16 years, since 2016; evaluation identifies parental consent, education, follow-up, counseling, and stigma as completion challenges (Tan et al., 2023). Iran has a longstanding premarital thalassemia screening model linked to counseling and prenatal diagnosis (Samavat and Modell, 2004). These programs must preserve informed choice, confidentiality, confirmation, and reproductive autonomy.

## 8 Closing the completion gap: a tiered LMIC model

The proposed tiered pathway, its principal attrition points, corrective enablers, and completion metrics are illustrated in Figure 1. Level 1, at primary or district facilities, should capture clinical and three-generation family history, recent transfusion and treatment, CBC and red-cell indices, smear, reticulocyte count where available, iron status when indicated, and region-appropriate low-cost or point-of-care screening. Level 2 should provide HPLC or CE, IEF in suitable newborn pathways, contextual review of age, pregnancy, iron status, transfusion and therapy, and orthogonal phenotyping for discordant patterns. Parental or other family samples should be used when segregation is informative, and partner testing should be used to clarify reproductive risk. Level 3 should provide regionally informed common-mutation panels, gap-PCR, MLPA, Sanger sequencing, or targeted NGS. Prenatal or preimplantation testing should proceed only after parental variants are securely characterized.

**FIGURE 1:**
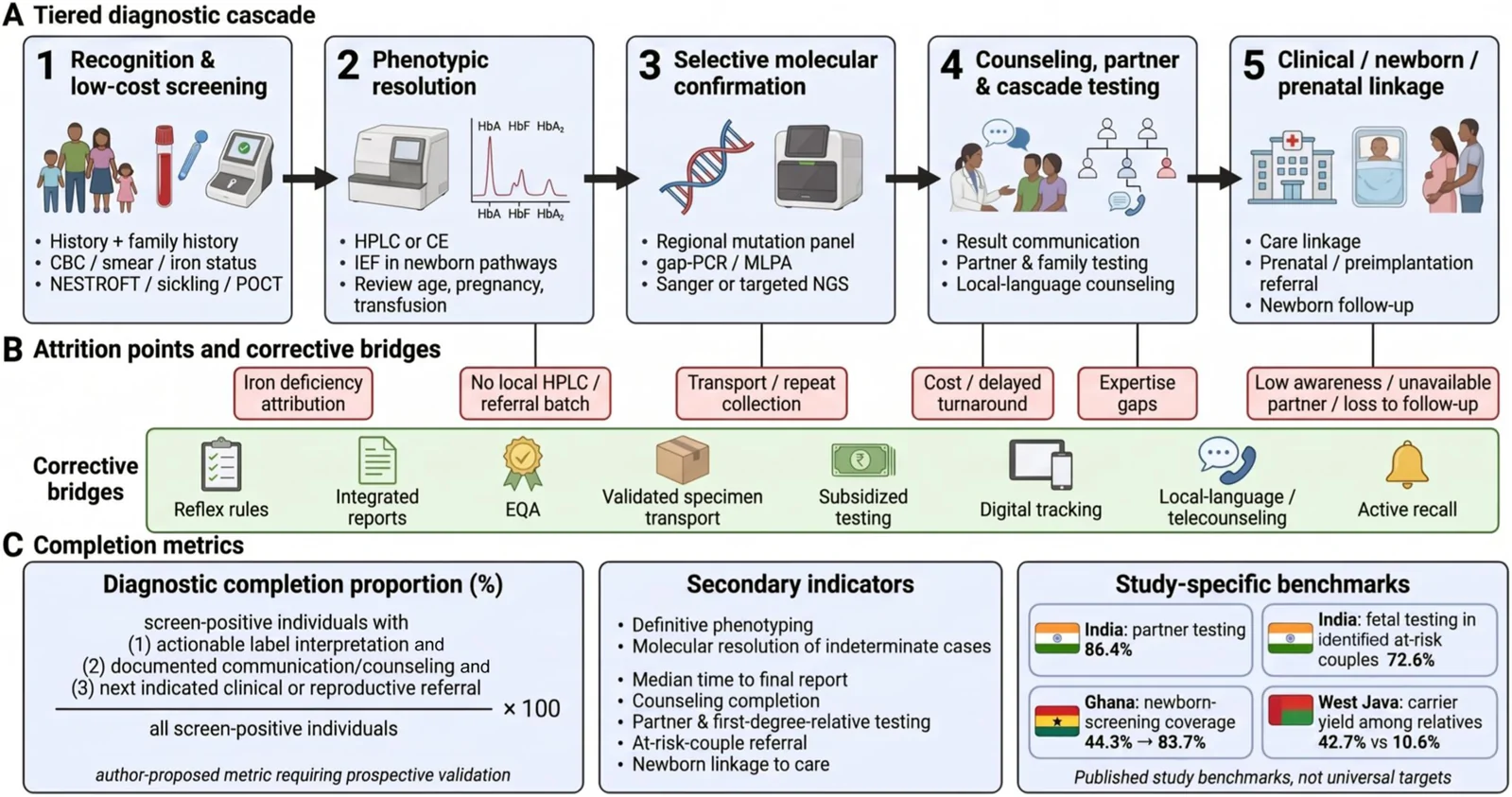
Proposed tiered diagnostic‐completion pathway for hemoglobinopathies in low- and middle-income countries. **(A)** The diagnostic cascade links primary/district recognition, regional phenotypic resolution, selective referral‐level molecular confirmation, counseling and family action, and clinical or reproductive linkage. **(B)** Attrition points at each handoff and corrective system enablers. **(C)** The proposed diagnostic completion proportion, secondary indicators, and published study‐specific benchmarks from India (Agarwal et al., 2025), Ghana (Segbefia et al., 2021), and West Java, Indonesia (Susanah et al., 2022). The diagnostic completion proportion is an author‐proposed metric that requires prospective validation; the study‐specific benchmarks are not universal targets. CBC, complete blood count; CE, capillary electrophoresis; EQA, external quality assessment; gap‐PCR, gap‐polymerase chain reaction; Hb, hemoglobin; HPLC, high‐performance liquid chromatography; IEF, isoelectric focusing; MLPA, multiplex ligation‐dependent probe amplification; NESTROFT, naked‐eye single‐tube red-cell osmotic fragility test; NGS, next‐generation sequencing; POCT, point‐of-care testing. Created in BioRender. Das, S. (2026) https://BioRender.com/f74ewjl.

Cross-cutting enablers are as important as instruments: standardized integrated reports, explicit referral rules, trained interpreters, external quality assessment, dried-blood-spot or validated specimen networks, subsidized testing, digital tracking, local-language counseling, telecounseling, and active recall. An integrated report should include age and indication, pregnancy and transfusion status, CBC and relevant iron results, platform and measured fractions, interpretive certainty, analytical limitations, the next test, and family or reproductive action.

Programs should move beyond counts screened. We propose the diagnostic completion proportion: the number of screen-positive individuals who receive (i) an actionable final interpretation, (ii) documented result communication and counseling, and (iii) the next indicated clinical or reproductive referral, divided by all screen-positive individuals. Complementary indicators should include definitive phenotyping after a positive screen, molecular resolution of indeterminate patterns, median time to final report, partner-testing uptake, first-degree-relative testing, fetal testing among eligible at-risk couples, and linkage of diagnosed newborns to care. This is an author-proposed program indicator that requires prospective validation.

Research priorities include cost-effectiveness of reflex algorithms, locally appropriate HbA2 thresholds, coverage of regional mutation panels, stage-specific attrition, counseling outcomes, partner and family uptake, confirmatory follow-up after newborn screening, and interoperability between laboratories and national registries.

## 9 Discussion and conclusion

Hemoglobinopathy diagnosis in LMICs is a clinical, laboratory, logistical, and counseling pathway rather than a sequence of isolated assays. Molecular analysis cannot correct incomplete history, poor-quality first-line testing, or uncritical interpretation of analyzer windows; it may merely transfer uncertainty to a more expensive platform. Conversely, universal sequencing is neither necessary nor feasible. Molecular confirmation should be directed to diagnostic ambiguity, complex genotype assignment, family segregation, and reproductive decision-making. This is particularly important for rare variants and compound states in which population screening identifies abnormality but cannot establish a clinically meaningful molecular identity.

The central weakness is often the handoff between available services. A 2026 survey of 38 centers in 20 countries documented marked variability in genomic access, screening arrangements, and laboratory policies (Stephanou et al., 2026). Country-level evidence locates the losses more precisely: 4% of women enrolled in a large Indian antenatal program did not complete initial testing, only 72.6% of identified at-risk couples completed fetal testing, and Ghanaian newborn-screening coverage at one tertiary site was initially 44.3% before workflow redesign (Agarwal et al., 2025; Segbefia et al., 2021). Screening volume alone is therefore an inadequate measure of success. A technically correct result that is not communicated, understood, and translated into partner testing, cascade screening, or care remains clinically and reproductively incomplete.

The evidence base is limited by heterogeneous platforms and thresholds, single-center designs, few prospective studies of diagnostic attrition, scarce analyses of cumulative pathway costs, and overreliance on case reports to illustrate adult delayed diagnosis. Regional data are not directly comparable because screening populations and denominators differ. The narrative selection of evidence and its concentration in South and Southeast Asia may introduce selection and geographical biases, limiting generalizability to other LMIC settings. Nevertheless, the convergent lesson is clear. Closing the hemoglobinopathy diagnostic gap in LMICs will require neither indiscriminate sequencing nor reliance on screening alone, but a tiered system that converts each abnormal result into an interpretable diagnosis, a communicated genetic risk, and an accessible next step for the individual and family.
