# Supplementary material for: Hemoglobinopathy diagnosis in LMICs: from hematological screening to molecular confirmation
Source: Front Genet. 2026 Nov 2;17:1969093. doi: 10.3389/fgene.2026.1969093 (PMC13770549; doi:10.3389/fgene.2026.1969093)
Supplement: Supplementary file 1 [file Table1.docx]

Supplementary Material 1
Structured Literature Search and Narrative Synthesis Methods

# 1. Review design

This Mini Review used a structured, critical narrative synthesis. It was designed to integrate laboratory diagnostic evidence with implementation, referral, financial, counseling, partner-testing, family-screening, and health-system evidence. It was not designed or reported as a systematic review, scoping review, or meta-analysis. No original patient data, unpublished institutional audit data, or personal communications were included. Statements that extend beyond directly reported findings are identified in the manuscript as author inferences or proposals.

# 2. Information sources and search date

The primary bibliographic database was PubMed/MEDLINE. The final search was conducted on 2 August 2026 and covered publications from 1 January 2020 through 2 August 2026. No language filter was applied at the search stage. No Embase, Scopus, or Web of Science search was undertaken; accordingly, this work should be interpreted as a structured narrative review rather than an exhaustive systematic review. Additional information sources comprised backward reference-list screening and targeted retrieval of official operational documents from the Government of India National Health Mission, the National Sickle Cell Portal, and the University of Ghana/Noguchi Memorial Institute for Medical Research. Journal websites were used to verify current article metadata when necessary.

# 3. Search concepts and terminology

| **Concept block** | **Principal MeSH headings** | **Free-text and title/abstract terms** |
| --- | --- | --- |
| **Disease and variant spectrum** | "Hemoglobinopathies"; "beta-Thalassemia"; "alpha-Thalassemia"; "Anemia, Sickle Cell" | hemoglobinopathy/haemoglobinopathy; thalassemia/thalassaemia; sickle cell disease/disorder; hemoglobin/haemoglobin variant; compound heterozygosity |
| **Hematological and phenotypic diagnosis** | "Erythrocyte Indices"; "Blood Cell Count"; "Reticulocytes"; "Chromatography, High Pressure Liquid"; "Hemoglobin Electrophoresis"; "Electrophoresis, Capillary" | CBC; MCV; MCH; RDW; smear; NESTROFT; solubility; sickling; HPLC; capillary electrophoresis; IEF; HbA2; borderline HbA2; co-elution |
| **Molecular diagnosis** | "Genetic Testing"; "Molecular Diagnostic Techniques" | molecular confirmation; genotyping; ARMS-PCR; allele-specific PCR; reverse dot blot; gap-PCR; MLPA; Sanger sequencing; next-generation sequencing; copy-number variation |
| **Access and diagnostic attrition** | "Delayed Diagnosis"; "Health Services Accessibility"; "Healthcare Disparities" | late diagnosis; diagnostic delay; referral; affordability; cost; sample transport; turnaround; loss to follow-up; diagnostic odyssey |
| **Family and reproductive action** | "Genetic Carrier Screening"; "Genetic Counseling"; "Prenatal Diagnosis"; "Neonatal Screening"; "Consanguinity" | partner testing; family screening; cascade screening; preconception; antenatal; prenatal; newborn; consanguinity; endogamy; stigma; disclosure |
| **LMIC and regional setting** | "Developing Countries"; "India"; "Asia, Southern"; "Africa South of the Sahara" | LMIC; resource-limited; India; Bangladesh; Sri Lanka; Thailand; Indonesia; Ghana; Nigeria; South Asia; Southeast Asia; sub-Saharan Africa |

# 4. Exact PubMed search strategy

The following strings are reported exactly in a reproducible Advanced Search format. Base blocks were entered as Searches #1-#3 in PubMed Advanced Search, followed by the thematic combinations shown below.

## Search #1: Disease block

("Hemoglobinopathies"[MeSH Terms] OR "beta-Thalassemia"[MeSH Terms] OR "alpha-Thalassemia"[MeSH Terms] OR "Anemia, Sickle Cell"[MeSH Terms] OR hemoglobinopath*[Title/Abstract] OR haemoglobinopath*[Title/Abstract] OR thalassemia*[Title/Abstract] OR thalassaemia*[Title/Abstract] OR "sickle cell disease"[Title/Abstract] OR "sickle cell disorder"[Title/Abstract] OR "sickle cell disorders"[Title/Abstract] OR "hemoglobin variant"[Title/Abstract] OR "hemoglobin variants"[Title/Abstract] OR "haemoglobin variant"[Title/Abstract] OR "haemoglobin variants"[Title/Abstract])

## Search #2: LMIC and regional block

("Developing Countries"[MeSH Terms] OR "low-income country"[Title/Abstract] OR "low-income countries"[Title/Abstract] OR "middle-income country"[Title/Abstract] OR "middle-income countries"[Title/Abstract] OR "low- and middle-income country"[Title/Abstract] OR "low- and middle-income countries"[Title/Abstract] OR "low and middle income country"[Title/Abstract] OR "low and middle income countries"[Title/Abstract] OR LMIC*[Title/Abstract] OR "resource-limited setting"[Title/Abstract] OR "resource-limited settings"[Title/Abstract] OR "resource-constrained setting"[Title/Abstract] OR "resource-constrained settings"[Title/Abstract] OR India[MeSH Terms] OR India[Title/Abstract] OR Bangladesh[Title/Abstract] OR "Sri Lanka"[Title/Abstract] OR Thailand[Title/Abstract] OR Indonesia[Title/Abstract] OR Ghana[Title/Abstract] OR Nigeria[Title/Abstract] OR "Asia, Southern"[MeSH Terms] OR "South Asia"[Title/Abstract] OR "Southeast Asia"[Title/Abstract] OR "Africa South of the Sahara"[MeSH Terms] OR "sub-Saharan Africa"[Title/Abstract])

## Search #3: Publication period

("2020/01/01"[Date - Publication] : "2026/08/02"[Date - Publication])

## Thematic Search 1: Overall diagnostic landscape

#1 AND #2 AND (diagnos*[Title/Abstract] OR screen*[Title/Abstract] OR "Mass Screening"[MeSH Terms] OR "Genetic Testing"[MeSH Terms] OR "Molecular Diagnostic Techniques"[MeSH Terms]) AND #3

## Thematic Search 2: Hematological screening and phenotypic methods

#1 AND #2 AND ("Erythrocyte Indices"[MeSH Terms] OR "Blood Cell Count"[MeSH Terms] OR "Reticulocytes"[MeSH Terms] OR "red cell indices"[Title/Abstract] OR CBC[Title/Abstract] OR microcyt*[Title/Abstract] OR "peripheral smear"[Title/Abstract] OR "blood film"[Title/Abstract] OR NESTROFT[Title/Abstract] OR "solubility test"[Title/Abstract] OR HPLC[Title/Abstract] OR "high performance liquid chromatography"[Title/Abstract] OR "capillary electrophoresis"[Title/Abstract] OR "hemoglobin electrophoresis"[Title/Abstract] OR "haemoglobin electrophoresis"[Title/Abstract]) AND #3

## Thematic Search 3: Molecular confirmation

#1 AND #2 AND ("Genetic Testing"[MeSH Terms] OR "Molecular Diagnostic Techniques"[MeSH Terms] OR "molecular confirmation"[Title/Abstract] OR genotyp*[Title/Abstract] OR "gap-PCR"[Title/Abstract] OR "ARMS-PCR"[Title/Abstract] OR "reverse dot blot"[Title/Abstract] OR MLPA[Title/Abstract] OR "Sanger sequencing"[Title/Abstract] OR "next-generation sequencing"[Title/Abstract] OR NGS[Title/Abstract]) AND #3

## Thematic Search 4: Diagnostic pitfalls

#1 AND ("borderline HbA2"[Title/Abstract] OR "borderline Hb A2"[Title/Abstract] OR "iron deficiency"[Title/Abstract] OR "alpha thalassemia"[Title/Abstract] OR "delta thalassemia"[Title/Abstract] OR transfus*[Title/Abstract] OR hydroxyurea[Title/Abstract] OR "rare variant"[Title/Abstract] OR "rare variants"[Title/Abstract] OR coelut*[Title/Abstract] OR coinherit*[Title/Abstract] OR co-inherit*[Title/Abstract] OR misclassif*[Title/Abstract]) AND (HPLC[Title/Abstract] OR electrophoresis[Title/Abstract] OR diagnos*[Title/Abstract]) AND #3

## Thematic Search 5: Delayed diagnosis, affordability, and follow-up

#1 AND #2 AND ("Delayed Diagnosis"[MeSH Terms] OR "late diagnosis"[Title/Abstract] OR "delayed diagnosis"[Title/Abstract] OR "diagnostic delay"[Title/Abstract] OR undiagnos*[Title/Abstract] OR misdiagnos*[Title/Abstract] OR "adult diagnosis"[Title/Abstract] OR adulthood[Title/Abstract] OR "Health Services Accessibility"[MeSH Terms] OR "Healthcare Disparities"[MeSH Terms] OR financ*[Title/Abstract] OR afford*[Title/Abstract] OR cost*[Title/Abstract] OR barrier*[Title/Abstract] OR follow-up[Title/Abstract] OR "lost to follow-up"[Title/Abstract] OR referral[Title/Abstract]) AND #3

## Thematic Search 6: Partner, family, and cascade screening

#1 AND #2 AND ("Genetic Carrier Screening"[MeSH Terms] OR "cascade screening"[Title/Abstract] OR "family screening"[Title/Abstract] OR "extended family screening"[Title/Abstract] OR "family testing"[Title/Abstract] OR "partner screening"[Title/Abstract] OR "partner testing"[Title/Abstract] OR "premarital screening"[Title/Abstract] OR "preconception screening"[Title/Abstract] OR "antenatal screening"[Title/Abstract]) AND #3

## Thematic Search 7: Counseling and sociocultural barriers

#1 AND #2 AND ("Genetic Counseling"[MeSH Terms] OR counsel*[Title/Abstract] OR psychosocial[Title/Abstract] OR stigma[Title/Abstract] OR acceptab*[Title/Abstract] OR "decision making"[Title/Abstract] OR autonomy[Title/Abstract]) AND #3

## Thematic Search 8: Consanguinity and endogamy

#1 AND #2 AND ("Consanguinity"[MeSH Terms] OR consanguin*[Title/Abstract] OR endogam*[Title/Abstract]) AND #3

## Thematic Search 9: Newborn, prenatal, and decentralized implementation

#1 AND #2 AND ("Neonatal Screening"[MeSH Terms] OR "newborn screening"[Title/Abstract] OR "Prenatal Diagnosis"[MeSH Terms] OR "antenatal screening"[Title/Abstract] OR "Point-of-Care Testing"[MeSH Terms] OR "point-of-care"[Title/Abstract] OR decentral*[Title/Abstract] OR implementation[Title/Abstract] OR registry[Title/Abstract] OR registries[Title/Abstract]) AND #3

## Thematic Search 10: India-specific referral, laboratory, and transport barriers

#1 AND (India[MeSH Terms] OR India[Title/Abstract]) AND (HPLC[Title/Abstract] OR "high performance liquid chromatography"[Title/Abstract] OR "molecular testing"[Title/Abstract] OR referral[Title/Abstract] OR transport*[Title/Abstract] OR centralis*[Title/Abstract] OR centraliz*[Title/Abstract] OR "hub-and-spoke"[Title/Abstract] OR cost*[Title/Abstract] OR laborator*[Title/Abstract]) AND #3

## Thematic Search 11: Ghana, Nigeria, and sub-Saharan African newborn-screening implementation

#1 AND ("Africa South of the Sahara"[MeSH Terms] OR "sub-Saharan Africa"[Title/Abstract] OR Ghana[Title/Abstract] OR Nigeria[Title/Abstract]) AND ("Neonatal Screening"[MeSH Terms] OR "newborn screening"[Title/Abstract] OR diagnos*[Title/Abstract] OR implementation[Title/Abstract] OR "dried blood spot"[Title/Abstract] OR IEF[Title/Abstract] OR HPLC[Title/Abstract] OR "point-of-care"[Title/Abstract] OR linkage[Title/Abstract] OR follow-up[Title/Abstract]) AND #3

# 5. Targeted official-source retrieval

Official sources were searched separately because national operational manuals, program dashboards, and reference-laboratory descriptions are incompletely indexed in PubMed. Targeted retrieval used the issuing institution and document title, followed by verification on the official domain. Sources retained for the manuscript included the Government of India National Health Mission hemoglobinopathy guidelines, the 2025 National Sickle Cell Application User Manual, and the University of Ghana/Noguchi Memorial Institute description of the national newborn-screening laboratory. Official sources were used for program architecture, operational definitions, and reported program totals, not as substitutes for peer-reviewed evidence of diagnostic accuracy or comparative effectiveness.

# 6. Evidence selection and prioritization

- Recent diagnostic reviews, method-comparison studies, laboratory studies, multicountry surveys, national or regional screening evaluations, newborn and antenatal screening studies, counseling research, family/cascade-screening studies, and implementation analyses were prioritized.
- Studies were retained when they directly informed screening performance, phenotype interpretation, molecular-referral criteria, diagnostic delay, referral or transport, financial access, partner/family testing, counseling, or linkage to care.
- Treatment-only, transfusion-management, chelation, transplantation, and gene-therapy publications were excluded unless they contained directly relevant diagnostic information.
- Case reports were used sparingly and only to illustrate adult or pregnancy-associated delayed diagnosis; they were not used to estimate prevalence or program performance.
- Country-level values were included only when the denominator and outcome were explicitly reported. Values from different study designs and populations were not treated as directly comparable national estimates.

# 7. Treatment of landmark pre-2020 sources

The 2020 date limit was used to prioritize current evidence. Earlier sources were retained selectively when they provided national policy, foundational regional mutation-distribution data, established diagnostic principles, remote-sampling evidence, or a program baseline not replaced by a newer source. Their role was identified contextually rather than combined indiscriminately with contemporary implementation data.

# 8. Data extraction and synthesis

For country and program comparisons, the authors extracted setting, study population, denominator, dominant variant pattern, diagnostic sequence, numerical screening or completion outcomes, and the principal point of attrition. Percentages were recalculated only from published numerators and denominators. The synthesis was organized around a diagnostic-completion cascade: recognition, phenotypic resolution, selective molecular confirmation, result communication and counseling, partner/family action, and clinical or reproductive linkage. The diagnostic completion gap, cumulative pathway cost, indeterminate hemoglobinopathy pattern, and diagnostic completion proportion are author-proposed interpretive constructs derived from this synthesis and require prospective validation.

# 9. Risk-of-bias and certainty assessment

No duplicate independent screening, formal study-level risk-of-bias assessment, or GRADE-style certainty rating was undertaken because the article is a focused Mini Review rather than a systematic review. This limits claims about comparative effectiveness and causal attribution. Conclusions were therefore restricted to convergent diagnostic and implementation patterns, while heterogeneous denominators, platforms, thresholds, and settings were explicitly acknowledged in the manuscript and Table 1.

# 10. Manuscript-ready search statement

PubMed/MEDLINE was searched on 3^rd^ July 2026 for literature published from 1 January 2020 to 2 August 2026 using MeSH and title/abstract terms relating to hemoglobinopathies, LMICs, hematological and phenotypic screening, molecular diagnosis, diagnostic delay, referral and affordability, genetic counseling, cascade screening, consanguinity, and newborn or prenatal implementation. No language restriction was applied. Recent diagnostic reviews, primary laboratory and implementation studies, and regional evidence with explicit denominators were prioritized. Earlier landmark sources were retained when they provided foundational regional epidemiology, national policy, or established diagnostic principles. The evidence was synthesized narratively; no formal study-level risk-of-bias or certainty assessment was undertaken.

# 11. Reproducibility statement

The exact PubMed strings, date range, evidence domains, and synthesis principles are reported above to permit replication or future updating. Search yields may change as PubMed indexing is updated. A future systematic or scoping review should add database expansion, duplicate screening, formal study selection, risk-of-bias assessment, and prospectively registered methods.
