# Supplementary material for: Hemoglobinopathy diagnosis in LMICs: from hematological screening to molecular confirmation
Source: Front Genet. 2026 Nov 2;17:1969093. doi: 10.3389/fgene.2026.1969093 (PMC13770549; doi:10.3389/fgene.2026.1969093)
Supplement: Supplementary file 2 [file Table2.docx]

**Supplementary Table 1**

**Diagnostic grey zones affecting HbA2 quantification and hemoglobin separation**

*Companion to: Hemoglobinopathy Diagnosis in LMICs: From Hematological Screening to Molecular Confirmation*

*Authors: Savitri M. Nerune; Sayandeep K. Das*

| **Scenario and expected analytical effect** | **Diagnostic risk** | **Recommended resolution** |
| --- | --- | --- |
| Iron deficiency anemia Usually lowers HbA2; the magnitude in beta-thalassemia carriers is inconsistent. | May mask mild or silent beta-thalassemia trait and create a false-negative or borderline result. | Assess ferritin or iron status; correct deficiency and repeat testing when the result is near the cut-off. Do not exclude carrier status from a low-normal HbA2 alone. (Colaco and Nadkarni, 2021; Thilakarathne et al., 2024) |
| Pregnancy A downward shift in the HbA2 reference interval has been reported in some populations. | A fixed non-pregnant cut-off may undercall a carrier or create an indeterminate result. | Use pregnancy- and platform-specific intervals; in an at-risk couple, proceed to partner testing and molecular resolution. (Kang et al., 2022) |
| Alpha- or delta-thalassemia / delta-globin variant HbA2 may be reduced, normalized, or divided between HbA2 and a separate delta-variant fraction. | Co-inherited beta-thalassemia may be masked. | Interpret CBC and family data; account for validated delta fractions and use segregation or molecular testing when reproductive risk is present. (Colaco and Nadkarni, 2021; Colaco et al., 2022; Thilakarathne et al., 2024) |
| Megaloblastic anemia HbA2 may increase and occasionally cross a beta-thalassemia carrier threshold. | May produce a false-positive or indeterminate beta-thalassemia trait result. | Correct vitamin B12 or folate deficiency and repeat HPLC; use molecular testing if reproductive decisions are time-sensitive. (Sahoo et al., 2023; Thilakarathne et al., 2024) |
| Hyperthyroidism or antiretroviral therapy HbA2 may rise; zidovudine- or stavudine-associated macrocytosis can produce borderline or elevated results. | May produce a false-positive beta-thalassemia carrier assignment. | Review thyroid status and medication history; repeat after correction or treatment change when appropriate, or confirm molecularly. (Thilakarathne et al., 2024) |
| Platform bias / HbS-containing samples HPLC and CE can report systematically different HbA2; HbS-related minor fractions may enlarge the A2 window on some cation-exchange systems. | May overcall HbS/beta-thalassemia or beta-thalassemia trait. | Correlate HbA, HbS, indices, therapy, and family samples; confirm with CE or electrophoresis, or HBB analysis. (Kaur et al., 2023; Sani et al., 2024; Yadav et al., 2026) |
| HbE, Hb Lepore, or HbD-Iran These variants can elute within the HbA2 window on some HPLC systems, creating a large apparent "A2" fraction. | May cause variant misidentification or erroneous HbA2 quantification. | Use peak size, shape, and retention time only as clues; apply an orthogonal separation method and molecular confirmation. (Kaur et al., 2023; Thaker et al., 2024) |
| Recent transfusion or infancy Donor fractions create mixed patterns; HbA2 is physiologically low and HbF high in infancy. | May produce a false-normal, mixed, or age-inappropriate interpretation. | Use a pre-transfusion sample, defer repeat testing when clinically appropriate, or use molecular analysis; apply newborn-specific algorithms. (Sani et al., 2024; Yadav et al., 2026) |

**Note.** *Effects are context-, population-, and platform-dependent and should not be treated as invariant biological rules. HbA2, hemoglobin A2; CE, capillary electrophoresis; HPLC, high-performance liquid chromatography. References cited in the table are listed in the main manuscript.*
